# Individual neural transfer function affects the prediction of subjective depth of focus

**DOI:** 10.1038/s41598-018-20344-x

**Published:** 2018-01-30

**Authors:** Alexander Leube, Tim Schilling, Arne Ohlendorf, David Kern, Alex G. Ochakovski, M. Dominik Fischer, Siegfried Wahl

**Affiliations:** 1 Institute for Ophthalmic Research, Eberhard Karls University Tuebingen, Elfriede-Aulhorn-Str. 7, Tuebingen, 72076 Germany; 2 Carl ZEISS Vision International GmbH, Turnstr. 27, Aalen, 73430 Germany; 3 University Eye Hospital, Centre for Ophthalmology, Eberhard Karls University of Tuebingen, Elfriede-Aulhorn-Str. 7, Tuebingen, 72076 Germany; 4 Nuffield Laboratory of Ophthalmology, University of Oxford, Oxford, United Kingdom

## Abstract

Attempts to accurately predict the depth of focus (DoF) based on objective metrics have failed so far. We investigated the effect of the individual neural transfer function (iNTF) on the quality of the prediction of the subjective DoF from objective wavefront measures. Subjective DoF was assessed in 22 participants using subjective through focus curves of visual acuity (VA). Objective defocus curves were calculated for visual Strehl metrics of the optical (VSOTFa) and the modulation transfer function as well as the point spread function. DoF was computed for residual lower order aberrations (rLoA) and incorporation of iNTF. Correlations between subjective and objective DoF did not reach significance, when a) standard metrics were used and b) rLoA were considered (r_*max*_ = 0.33, p_*all*_ > 0.05). By incorporating the iNTF of the individuals in the calculation of the objective DoF from the VSOTFa metric, a moderate statistically significant correlation was found (r = 0.43, p < 0.01, Pearson). The iNTF of the individual’s eye is fundamental for the prediction of subjective DoF using the VSOTFa metric. Individualized predictions could aid future application in the correction of refractive errors like presbyopia using intraocular lenses.

## Introduction

The neural transfer function (NTF) is next to the optical transfer function (OTF) one part of the overall contrast sensitivity function of the human eye^1^. The assessment of the NTF requires to by-pass the optical part and to assess the perceptual part alone. Technically this can be achieved by using interference fringe technique^2,3^. While using this technique, a sinusoidal strip pattern is projected onto the retina that is not influenced by the optics of the eye. The contrast and the spatial frequency can be changed in a way that the neural part of the contrast perception (the neural transfer function) of the retina-brain-system, can be assessed separately from the characteristics of the optics. Using a second method, that measure the wavefront errors of an individual’s eye, the neural part of the perception of contrast gratings can be calculated^4^ out of the derived modulation transfer function (MTF) and the contrast sensitivity function (CSF). The knowledge of the neural contrast transfer property of the human visual system is an important parameter since image quality depends on both, the optical and the neural factors of vision^2,5–7^. Numerical expressions, the so called image quality metrics, consider both information, the objective measures of the optical quality and the psychophysically assessed neural perception function^7^. In 1987, Barten^8^ proposed a metric called “square root integral” (SQRI) that incorporated the neural threshold level of the human eye, with the aim to quantify the resolution visibility in displays. Later, Thibos *et al*.^5^ described 33 image quality metrics and showed high precision of wavefront based metrics for the prediction of the subjective spherical equivalent error.

Objective image quality metrics can also be used to calculate defocus curves and estimate the objective depth of focus at a defined threshold level^9^. So far, fixed threshold levels (for example 50%^10,11^ or 80%^12^) from the maximum of the defocus curve or individual thresholds that are based on the root mean square of the amount of higher order aberrations^9^, were used. The depth of focus of the eye is defined as “the greatest range of dioptric focusing error which does not result in objectionable deterioration in the retinal image quality”^13^. This definition includes that the subjective and objective measures strongly depend on the definition of an “objectionable deterioration” of the retinal image. However, some studies^14,15^ that tried to find good predictions of the subjective depth of focus calculated from image quality metrics failed. Nevertheless, the calculation of the depth of focus from objective through focus curve generated using image quality metrics is widely used for performance measure, for example of intraocular lenses^16–18^ or to investigate the influence of aberrations on the visual performance of the eye (for example in form of the visual acuity)^19–21^. One step further for the future of individualized biomedical applications is the use of personal data, such as the individual neural transfer function (iNTF).

Therefore, it was the aim of the current study to evaluate the influence of the iNTF and several confounding factors on the prediction of the subjective depth of focus from objective metrics.

## Results

In total, data of 22 participants with a mean age of 26.2 ± 3.1 years and a mean spherical equivalent refractive error (M) of −1.09 ± 2.39 D and a straight astigmatic component of *J*_0_ = 0.11 ± 0.34 D were included in the analysis.

### Calculation of objective depth of focus from standard parameter

The mean subjective DoF evaluated from the defocus curve (mean threshold level: −0.01 ± 0.10 logMAR) was 0.84 ± 0.27 D. Objective calculations for a pupil size of d = 4 mm gave significant lower values when the 80% threshold was considered (DoF_*VSOTFa*_: 0.37 ± 0.14 D, p < 0.001; DoF_*VSPSF*_: 0.35 ± 0.08 D, p < 0.001; DoF_*VSMTF*_: 0.40 ± 0.14 D, p < 0.001). DoF evaluated at the 50% threshold did not result in a significant difference for all the objective metrics calculated for a 4 mm pupil size (DoF_*VSOTFa*_: 0.75 ± 0.20 D, p > 0.05; DoF_*VSPSF*_: 0.73 ± 0.23 D, p > 0.05; DoF_*VSMTF*_: 0.86 ± 0.28 D, p > 0.05). By considering the default parameters and a 50% threshold level, the computed correlations for each metric were low (r_*VSOTFa*_ = 0.114, r_*VSPSF*_ = 0.174, r_*VSMTF*_ = 0.163, p_*all*_ > 0.05) and therefore no prediction of subjective depth of focus from objective visual Strehl metrics using default settings could be achieved.

### Parameter A - The influence of residual lower order aberrations

**Figure 1:**
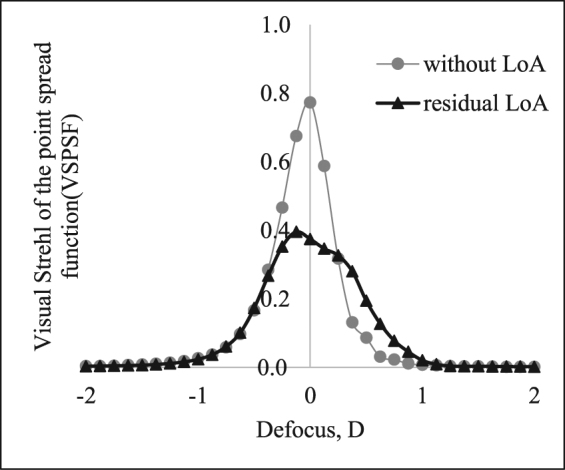
Comparison of defocus curves calculated without lower order aberrations (LoA) and with residual LoAs for a sample participant.

The correction of lower order aberrations (LoA) using trial lenses did not compensate all the errors of defocus and primary astigmatism due to the step size of 0.25 D that limits the precision of the subjective refraction. The calculation of the inaccuracy resulted in a mean residual LoA of 0.018 ± 0.155 *μ* m for defocus ${Z}_{2}^{0}$ and of 0.003 ± 0.061 *μ* m, −0.012 ± 0.060 *μ* m for oblique ${Z}_{2}^{-2}$ and straight ${Z}_{2}^{2}$ astigmatism, respectively. Mean values of residual LoAs were nearly zero, but standard deviation showed that there was a wide range of uncorrected lower order aberrations. Figure 1 shows a comparison of defocus curves from one participant, when LoAs were set to zero (gray) and when residual LoAs were considered (black). As expected, the peak value was lower in case the residual LoAs were taken into account. Because of the asymmetry of the astigmatism, the calculations of the defocus curve can lead to asymmetric shapes that will affect the calculation of the depth of focus, see Fig. 1. The mean objective DoF considering the residual astigmatism, was 0.67 ± 0.12 D for the VSOTFa, 0.74 ± 0.16 D for the VSPSF and 0.86 ± 0.20 D for the VSMTF metric. However, the recalculated defocus curves and the depth of focus for a 4 mm pupil diameter and a 50% threshold definition showed no significant correlations between subjective and objective measures (r _*VSOTFa*_ = 0.327, r_*VSPSF*_ = 0.085, r_*VSMTF*_ = 0.086, p_*all*_ > 0.05).

### Parameter B–The influence of the individual neural transfer function (iNTF)

**Figure 2:**
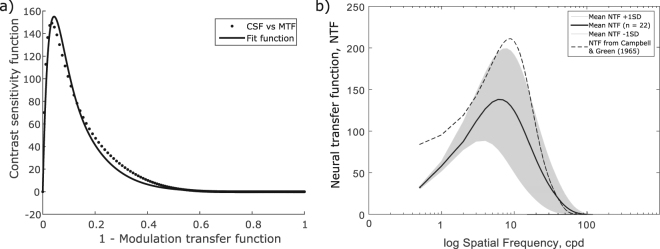
(**a**) Relationship between contrast sensitivity function (CSF) and modulation transfer function (MTF). Data was fitted with a double exponential square root function (Equation 1) and represents the model of the neural transfer function (NTF). (**b**) Comparison of mean neural transfer function from the study group (n = 22) ±*SD* and the NTF adapted from Campbell & Green^2^.

The objective image quality metric VSMTF was not calculated using iNTF because the calculation of the NTF was based on the MTF that would be also used in the calculation of the VSMTF. The neural transfer function describes the fraction of the contrast sensitivity function and the modulation transfer function. To model the unknown individual neural transfer function (iNTF) for the relationship between the CSF as an output and the MTF as an optical filter of the image, we investigated respective values from both, the CSF and the MTF, for the given spatial frequencies. Figure 2a shows that this relationship is non-linear and can be modeled by a double exponential square root function, see Equation 1 with the parameter a = 9725 and b = 25 (*R*^2^ = 0.95) for an individual participant. It reflects the neural contrast gain of the visual system. Compared to the standard NTF from Campbell and Green^2^, the mean NTF calculated from our study group (n = 22) showed a shifted maximum towards lower spatial frequencies and a lower peak value of 130.7 ± 58.8 at 6.0 cpd, Fig. 2b. It is given by Equation 1 with the parameter a = 59.12 and b = 0.17.

1 $$NTF(sf)=a\ast sf\ast {e}^{-b\ast sf}\ast \sqrt{1+\mathrm{0.06\ast }{e}^{b\ast sf}}$$

**Figure 3:**
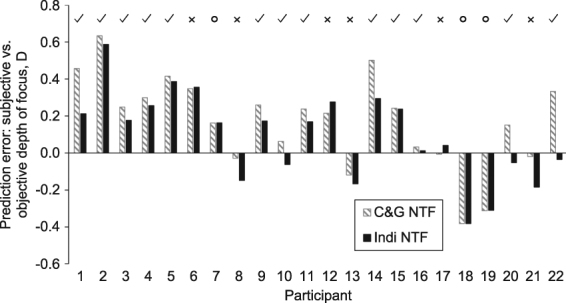
Individual differences between subjective and objective DoF (D) for through focus analysis using the C&G NTF^2^ and the individual NTF. Smaller prediction errors are indicated with ✓, whereas no change is shown with ° and higher errors are indicated by X.

Results of the calculations showed a wide range of individual differences in the iNTF. Objective defocus curves were calculated for VSOTFa and VSPSF, using a 4 mm pupil diameter, the individual neural transfer function and the incorporation of residual LoA. Mean objective DoF was 0.75 ± 0.15 D for the VSOTFa and 0.80 ± 0.17 D for the VSPSF metric. The correlations with the subjectively measured DoF were moderate for both metrics (r _*VSPSF*_ = 0.212, p_*VSPSF*_ = 0.33) and a statistically significant correlation was found between the objective DoF calculated using the VSOTFa and the subjective depth of focus (*DoF*_*subj*_ = 0.74*DoF_*VSOTFa*_ + 0.28, r = 0.431, p = 0.04, Pearson) by including iNTF in the calculations of the objective DoF. However, regression analysis revealed a moderate prediction (*R*^2^ = 0.19) of the subjective depth of focus. Evaluating the individual differences with and without the individual NTF provides a similar picture (Fig. 3). The mean difference between the subjective and the objective depth of focus using the standard NTF was 0.16 ± 0.29 D, whereas the mean difference using the iNTF was significant smaller 0.08 ± 0.28 D (p < 0.001, t-test).

## Discussion

### Objective depth of focus and image quality metrics

The prediction of subjective depth of focus (DoF) from a single wavefront measurement has a big influence on the research in vision science. Objective assessed DoF in the current study is comparable to earlier reported values: Marcos *et al*.^12^ found DoF of 0.4 D for double-pass images and 1.6 times smaller values from wavefront simulations of the modulation transfer function. They further revealed a discrepancy between the objectively and subjectively assessed DoF^12^ as it ruled out in the current study using the default parameter. In the current study, the assessment of subjective depth of focus was based on an objective blur criteria (*VA*_*max*_ + 0.1*logMAR*), which is free of individual interpretations of instructions on blur perception^22^. However, the use of a Badal system would enable a measurement with smaller steps size of the induced defocus, but requires a subjective blur criteria.

Yi *et al*.^9^ showed that the objective depth of focus can be calculated from through focus curves of image quality metrics for fixed thresholds^11,12^ and estimated the objective DoF as 1.07D for a 50% threshold and as 0.52D for a 80% threshold. The slightly higher values originate from the difference in pupil size, since they used a pupil diameter of 3.5 mm. They further introduced a method to calculate individual thresholds based on the root mean square of the coefficients of higher order aberrations (RMS-HOA). This method provides a higher weighting of HOA than using fixed thresholds. Nevertheless, their study gave no information about correlations between subjective and objective depth of focus. Legras *et al*.^14,15^ investigated different definitions of objective image quality metrics (different optical parameter and spatial frequency ranges) and their impact on the prediction of subjective depth of focus, measured with adaptive optics. The correlation coefficients were dependent on the letter size that was used for the subjective measurements. For a letter size of 0.1 logMAR (mean threshold level in the present study was −0.01 ± 0.10 logMAR), the regression coefficients (*R*^2^) ranged between < 0.01 and 0.1. This is in accordance with the current data, when the default settings (4 mm pupil size, LoA = 0 and NTF from Campbell & Green^2^) were used. The low correlation values found in the present study using the default parameters could be explained by the missing correlation between maximum visual acuity and maximum metric value (*R*^2^ < 0.1, Pearson, for all three VS-metrics). Villegas *et al*.^23^ did not find a significant correlation between visual acuity (VA) and optical quality calculated from image quality metrics in young subjects with normal or excellent vision. It was also shown that the prediction of VA from optical quality metrics increases in low luminance condition, but is poor for bright lighting conditions^24^, which might give a hint that quality metrics are not suitable for high performance optical systems. Contrariwise, Marsack *et al*.^20^ found that the visual Strehl metric from the OTF can account for 81% of the average variance in high-contrast logMAR visual acuity measurements, based on three individuals.

### Pupil size dependency

There is a strong dependency of the depth of focus (DoF) on the pupil size. A smaller pupil size will produce a larger depth of focus^12,25^. DoF calculated from image quality metrics does not show this dependency^9^. Furthermore, Marcos *et al*.^12^ showed that the objective DoF assessed by double-pass-imaging or wavefront simulations follows a non-monotonic function as the pupil size increases. The results from the current study were therefore evaluated for a fixed pupil diameter of 4 mm, for both, the objective and the subjective measurements. An increases in pupil size would lead to a higher optical vergence in the pupil’s plane. Benard *et al*.^14^ found a good correlation between the induced variation of vergence within the full pupil diameter and subjectively measured depth of focus. On the other hand, the Stiles-Crawford effect^26^ (the luminous efficiency of the eye decrease as more off axis the light passes the pupil) limits the effect of the impact of optical vergence on the depth of focus. Furthermore, under natural viewing conditions, the subject’s pupil diameter alters and is not fixed. Buehren *et al*.^27^ demonstrated that the correlation coefficients between visual acuity and VSOTFa calculated from higher order aberrations becomes higher when the natural pupil diameter is considered (r = 0.56, p < 0.1) while correlations using a fixed 3 mm pupil diameter were worse (r = 0.43, p > 0.1).

### Monochromatic and polychromatic aberrations

Calculations of residual lower order aberrations and their incorporation into the evaluation of the depth of focus from objective defocus curves resulted in asymmetric shapes (Fig. 1). One can expect that this asymmetry causes an increase of the individual variations of the depth of focus, when the DoF is predicted from image quality metrics using objective defocus curves. But these correlation coefficients did not changed by taking residual LoAs into account, an observation that was supported by Yi *et al*.^9^ The authors reported that the correlation of the estimated VSOTF threshold and the RMS-HOA dropped down, when the residual astigmatism was considered. Results from Thibos *et al*.^28^ showed that a subjective refraction via optimized visual acuity is not based on the criterion of minimizing the wavefront variance^29^ like it is done by setting the lower order aberrations to zero. The used method from the current study to estimate residual lower order aberration by correcting the Zernike terms by the sign-switch coefficients from the subjective refraction could be disadvantageous. The usage of adaptive optics systems^30^ to correct lower and higher order aberrations would be preferred in future studies to investigate the neural involvement^31^ and the effect of lower order aberrations on the depth of focus.

All calculations of optical quality parameters were performed for monochromatic light and a reference wavelength of *λ* = 550 nm, according to the eye’s photopic spectral sensitivity. Because aberrations are a function of the wavelength (see also the section “From wavefront data to the depth of focus” in the methods), the presented results will be different if the reference wavelength is changed. Subjective measures were performed in polychromatic white light conditions. Recalculation of metric values using polychromatic point spread function^32^ is assumed to lead to different through focus curves from objective image quality metrics and to a change in correlations between objective and subjective depth of focus. The range of the focal shift associated with the longitudinal chromatic aberration^33^ weighed by photopic spectral sensitivity of the eye would increase the calculated depth of focus^12,32^. Contrariwise, Jaskulski *et al*.^34^ revealed that there is no significant effect of LCA and the depth of focus comparing subjective measures from green and white light. However, from the results of the current study we can not conclude whether polychromatic image quality simulations influence the prediction of subjective depth of focus.

### Individual neural transfer function iNTF

Calculating the neural transfer function (NTF) as the fraction between the contrast sensitivity function (CSF) and the modulation transfer function (MTF) was shown to provide good predictability regarding the visual acuity^1^ and can serve as good approximation for the NTF. However, a more precise and straightforward assessment of the NTF would be to use interference fringes^2,35^. The comparison of the NTF measured with interference fringes by Campbell & Green^2^ and the calculated NTF from current study showed that the calculations result in lower NTF’s. This is explained by the fact that using interference fringes by passes the eye’s optic dynamically and is furthermore a diffraction limited measurement. The incorporation of individual calculated NTF into the VSOTFa metric leads to an increase of the correlation coefficient to r = 0.43, with the result that the subjective depth of focus can be predicted from the objective measures. The increase in individual information which is provided for metric calculations by the additional individual NTFs produce an expectable enhancement of the goodness of the correlation. Individual neural transfer function gives similar measures of contrast transfer as the OTF, since both characterize the modulation of contrast for different spatial information of an optical system. On the contrary, visual acuity estimates a threshold level of the smallest resolvable spatial detail and is defined as the cutoff frequency. This fundamental difference in measures leads to challenging problems while comparing the area under the optical transfer functions and a single acuity value. For the depth of focus, the estimation and judgment of blur, the contrast of the perceived image seems to play a major role.

The present study investigated how the correlation between objective DoF estimations from image quality metrics can provide moderate predictions for subjective DoF. Calculations from the VSOTFa metric resulted in superior correlation when compared to the VSPSF and VSMTF metric. However, the parameter residual lower order aberrations do not explain the major variance of subjective measures. For the prediction of subjective DoF using the VSOTFa metric, the CSF of the individual eye plays a major role and its incorporation using the iNTF into the visual Strehl metrics results in a moderate, significant correlation between the objective and the subjective DoF. The results of the study could enable scientists as well as industry in ophthalmology and vision science to develop individualized solutions for a better performance of intraocular lenses or vision after refractive surgery in the future. This will improve current methods that only take standardized measures into account.

## Methods

### Participants

Twenty eight young and eye healthy participants were enrolled in the study. Six participants had to be excluded from the analysis because of multimodal defocus curves, which would result in discontinuities of the depth of focus estimations. The following inclusion criteria were checked during an ophthalmological examination before the start of the study: visual acuity greater or equal to 0.0 logMAR, ametropia in the dominate eye lower than ± 6.0 DS and ± 2.0 DC and no known eye diseases. Participants were excluded from the study if they suffered from allergy to Cyclopentolate Hydrochloride or showed contradictions to mydriasis. Prior to the experiment, accommodation was blocked using three drops of 1% Cyclopentolate Hydrochloride with a time duration of 30 minutes within 10 minutes in between every application. Objective refraction of the errors of the eye was achieved using a wavefront aberrometer (ZEISS i.Profiler plus, Carl Zeiss Vision GmbH, Germany) and the measurements were repeated three times to account for fluctuations of tear film and residual accommodation. The most positive reading of the objective measures were used as starting values to perform subjective distance refraction at 5 m using a single line of SLOAN letter optotypes^36^. The end point of the monocular refraction was the maximum plus power with the highest visual acuity. Both refraction procedures, as well as all study-related measurements, were obtained after full cycoplegic effect emerged. To control accommodation paralysis, two push-up measurements from clear to first noticeable blur in distal and proximal distance were performed. Participants with an accommodation range higher than 1 D after cycloplegia were excluded from the analysis. The study followed the tenets of the Declaration of Helsinki and was approved by the Institutional Review Board of the medical faculty of the University of Tuebingen. Informed Consent was obtained from all participants after the content and possible consequences of the study had been explained.

### Protocol

#### Assessment of subjective depth of focus

Participants wore their distance correction in a trial frame (UB4, Oculus, Germany) corrected for a viewing distance of 5 m. To evaluate the subjective depth of focus, defocus curves of high contrast visual acuity in a dioptric range of ± 1.50 D within 0.5 D steps were measured. The method to calculate the depth of focus from subjective obtained defocus curve was described elsewhere^37^. In brief, DoF is defined as the horizontal dioptric range at the threshold level of + 0.1 logMAR below the maximum value of visual acuity. By normalizing the threshold level to the maximum acuity value, shifts in best performance position of the defocus curve were compensated. Luminance was controlled and set to photopic light conditions of L = 250 *cd*/*m*^2^. The assessment of the subjective depth of focus was performed under full cycoplegic conditions.

#### Contrast sensitivity and neural transfer function

To evaluate the contrast sensitivity^38^, a psychophysical staircase procedure (PSI Ψ method) was programmed in Matlab (Matlab 2014a, MathWorks Inc., Natick, USA) using the Palamedes Toolbox^39^. Stimuli were presented on a 120 Hz LCD display (VIEWPixx /3D, VPixx Technologies, Canada) with a 16 bit gray level resolution for each pixel. Mean luminance of the visual display was 65 *cd*/*m*^2^ and room illumination was reduced. Prior to the measurements, the participants were light adapted for at least 10 minutes. During a four-alternative forced choice (4AFC) task, the participant was asked to respond the direction (0°, 45°, 90° or 135°) of a sinusoidal grating (Gabor Patch). The grating was enveloped with a Gaussian filter function according to Equation 2.

2 $$I(x,y)={I}_{0}({\rm{\sin }}\mathrm{(2}\pi f[y{\rm{\sin }}(\theta )+x{\rm{\cos }}(\theta )])\ast {e}^{\frac{({x}^{2}+{y}^{2})}{2{{\rm{\sigma }}}^{2}}})$$

The gray scale intensity of each pixel I(x,y) in the x,y position of the screen was defined by the mean gray level *I*_0_, the frequency of a sine wave f (1/pixel) and an angular tilt *θ*. Stimulus size was set to 2.5° of visual angle with a Gaussian sigma of *σ* = 0.1° for a viewing distance of 5 m. To achieve reliable measures for the smallest detectable contrast at each spatial frequency (from 0.5 to 60 cpd in 14 log-steps: 0.5, 1.0, 2.0, 3.0, 5.0, 7.0, 9.0, 11.0, 13.0, 15.0, 23.0, 32.0, 44.0 and 60.0 cpd), 50 trials of stimuli presentation in the adaptive staircase procedure starting with an initial Michelson contrast of 0.55 were performed. All measurements were done under cycloplegic, monocular conditions in the dominat eye while a 4 mm artificial pupil was placed in front of the participant’s eye. The measured contrast sensitivity data was fitted with a double exponential function, adapted from Barten^7^, to reduce the noise of the measurement. Individual neural transfer functions (iNTF), which require next to the CSF the optical transfer function of the eye, were implemented into image quality metrics (Equations 5–7) to calculate the objective depth of focus.

### From wavefront data to the depth of focus

The method to calculate defocus curves based on wavefront measurements and derive depth of focus was described by Yi *et al*.^9^ Optical measurements of wavefront aberrations were performed since full cycloplegic effect emerged. Standardized Zernike coefficients from the aberrometer measurement were used to reconstruct the wavefront by summing the Zernike polynomials weighted with the coefficients. The point spread function (PSF) was calculated from the reconstructed wavefront as the Fourier transform of the pupil function. The optical transfer function (OTF) is defined by the Fourier transform of the PSF^40^ and refers to the direction and spatial specific properties of an optical system. It further contains the modulation transfer function (MTF = abs(OTF)) as well as the phase transfer function (PTF = angle(OTF)) as a complex numbered function. A Gaussian apodization filter including a gamma of *γ* = 0.115 mm^−2^ was used to model the Stiles-Crawford effect^5^. The transfer function between object contrast and perceived contrast in the human visual systems (contrast sensitivity function, CSF) is not fully described by the optical transfer properties of the eye. A neural transfer function (NTF) describes the modulations in the perception of contrast for the retina-brain system^2^ and has to be considered as well. The entire contrast sensitivity function can be modeled by the product of the optical and the neural transfer function^1^. Using Equation 3, the neural transfer function of the human visual system can be calculated^4^.

3 $$NTF({f}_{x},{f}_{y})=\frac{CSF({f}_{x},{f}_{y})}{MTF({f}_{x},{f}_{y})}$$

4 $$N(x,y)={ {\mathcal F} }^{-1}(NTF({f}_{x},{f}_{y}))$$

5 $$VSPSF=\frac{{\int }_{-\infty }^{\infty }N(x,y)\ast PSF(x,y)dxdy}{{\int }_{-\infty }^{\infty }N(x,y)\ast PS{F}_{DL}(x,y)dxdy}$$

6 $$VSOTFa=\frac{{\int }_{-\infty }^{\infty }NTF({f}_{x},{f}_{y})\ast \Re (OTF({f}_{x},{f}_{y}))d{f}_{x}d{f}_{y}}{{\int }_{-\infty }^{\infty }NTF({f}_{x},{f}_{y})\ast OT{F}_{DL}({f}_{x},{f}_{y})d{f}_{x}d{f}_{y}}$$

7 $$VSMTF=\frac{{\int }_{-\infty }^{\infty }NTF({f}_{x},{f}_{y})\ast MTF({f}_{x},{f}_{y})d{f}_{x}d{f}_{y}}{{\int }_{-\infty }^{\infty }NTF({f}_{x},{f}_{y})\ast MT{F}_{DL}({f}_{x},{f}_{y})d{f}_{x}d{f}_{y}}$$

In the present study, we evaluated visual Strehl metrics^5^ that are based on the optical transfer function (VSOTFa), the modulation transfer function (VSMTF) and the point spread function (VSPSF), where *f*_*x*_ and *f*_*y*_ are the spatial frequencies, *x* and *y* are the space coordinates and *DL* describes the diffraction limited function. For the VSOTFa, we used the augmented version proposed by Iskander^41^. Visual Strehl metrics compute the optical quality normalized to diffraction limited optics and weighted by the neural transfer function (see Equations 5, 6 and 7). Prior to the calculation of defocus curves, the measured wavefront data was scaled^42^ to a fixed pupil diameter of d = 4 mm and all necessary defocus steps (from −2.0 to + 2.0 in 0.125 D steps) were converted from the refractive domain to the wavefront domain. Calculations were performed with Matlab using a reference wavelength of *λ* = 550 nm and a spatial resolution of 2^7^ bit. Spatial frequencies ranged from 0 to 60 cycle/deg (cpd). Defocus curves and respectively the objective depth of focus were calculated for three different configurations: (1) default setting: Pupil diameter d = 4 mm, standard NTF^2^ and setting the low order aberrations (defocus ${Z}_{2}^{0}$ and astigmatism ${Z}_{2}^{-2},{Z}_{2}^{2}$) to zero. To account for the influence of residual lower order aberration (Parameter A), the second (2) configuration included a pupil diameter of d = 4 mm, the standard NTF^2^ and objective Zernike terms for defocus and for primary astigmatism were corrected with the sign-switched (from error to correction) Zernike coefficients from subjective refraction. Since contrast sensitivity plays a major role for calculating visual Strehl metrics, we have incorporated in configuration (3) the individual neural transfer functions iNTF (see Equation 3) in the metric calculations (Parameter B) and re-evaluated the correlation of the subjective depth of focus and the objective defocus curves for a pupil diameter of d = 4 mm and residual lower order aberrations. The iNTF data was fitted with a double exponential square root function adopted from Barten^7^ (see Equation 1).

**Figure 4:**
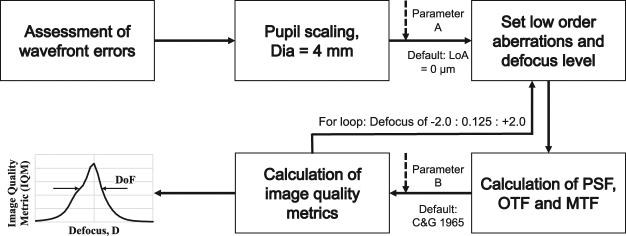
Workflow for calculation of through focus curve from wavefront errors using image quality metrics. The parameters A and B describe the residual low order aberrations (LoA) and the individual neural transfer function, respectively, that are evaluated regarding the DoF prediction. Dia = Pupil diameter.

To calculate the depth of focus from the defocus curves, we used threshold levels of 50% from the maximum metric value for all three visual Strehl metrics. The workflow of the calculations is shown in Fig. 4. In the standard definition of the visual Strehl metrics, the neural transfer function (NTF) from the publication of Campbell & Green^2^ is used. In the present study, we have evaluated the NTF of the individual participants and incorporated their individual data in the calculations of the defocus curves from image quality metrics.
